# Why Do We Shiver?

**Published:** 1893-02-11

**Authors:** 


### WHY DO WE SHIVER?

M. Richet has been studying the physiology of a shiver. He says, "If the temperature of a dog is lowered by any means whatever, for instance, by slowing his respirations by means of chloral, at the end of a certain time the temperature is seen to rise. It is then that the shiver appears and the quicker he regains heat the more violently he shivers. An energetic shiver, then, is a necessary part of regaining heat, and is one of the causes of the increase of combustion."

